# Machine Learning in Preoperative Prediction of Postoperative Immediate Remission of Histology-Positive Cushing’s Disease

**DOI:** 10.3389/fendo.2021.635795

**Published:** 2021-03-02

**Authors:** Wentai Zhang, Mengke Sun, Yanghua Fan, He Wang, Ming Feng, Shaohua Zhou, Renzhi Wang

**Affiliations:** ^1^ Department of Neurosurgery, Peking Union Medical College Hospital, Chinese Academy of Medical Sciences and Peking Union Medical College, Beijing, China; ^2^ Medical Imaging, Robotics, Analytic Computing Laboratory/Engineering (MIRACLE), Key Laboratory of Intelligent Information Processing of Chinese Academy of Sciences (CAS), Institute of Computing Technology, CAS, Beijing, China; ^3^ University of Chinese Academy of Sciences, Beijing, China

**Keywords:** Cushing’s disease, machine learning, transsphenoidal surgery, preoperative prediction, immediate remission

## Abstract

**Background:**

There are no established accurate models that use machine learning (ML) methods to preoperatively predict immediate remission after transsphenoidal surgery (TSS) in patients diagnosed with histology-positive Cushing’s disease (CD).

**Purpose:**

Our current study aims to devise and assess an ML-based model to preoperatively predict immediate remission after TSS in patients with CD.

**Methods:**

A total of 1,045 participants with CD who received TSS at Peking Union Medical College Hospital in a 20-year period (between February 2000 and September 2019) were enrolled in the present study. In total nine ML classifiers were applied to construct models for the preoperative prediction of immediate remission with preoperative factors. The area under the receiver operating characteristic (ROC) curve (AUC) was used to evaluate the performance of the models. The performance of each ML-based model was evaluated in terms of AUC.

**Results:**

The overall immediate remission rate was 73.3% (766/1045). First operation (p<0.001), cavernous sinus invasion on preoperative MRI(p<0.001), tumour size (p<0.001), preoperative ACTH (p=0.008), and disease duration (p=0.010) were significantly related to immediate remission on logistic univariate analysis. The AUCs of the models ranged between 0.664 and 0.743. The highest AUC, i.e., the best performance, was 0.743, which was achieved by stacking ensemble method with four factors: first operation, cavernous sinus invasion on preoperative MRI, tumour size and preoperative ACTH.

**Conclusion:**

We developed a readily available ML-based model for the preoperative prediction of immediate remission in patients with CD.

## Introduction

Pituitary adrenocorticotropic hormone (ACTH) hypersecretion, also known as Cushing’s disease (CD), is one of the aetiologies of Cushing syndrome(CS), causing a variety of manifestations such as fatigue, weight gain, osteoporosis, diabetes mellitus, thin skin, and ecchymoses (1). According to a consensus statement, the first-line treatment option for CD is transsphenoidal surgery (TSS) (2). In a systemic review including 2,614 CD patients who had undergone TSS, the overall remission rate was 77% (52.1%–96.6%) (3), and recurrence rates were in the range of 3%–66% (4, 5).

While several studies have revealed that postoperative hypocortisolaemia (6–8),low urinary free cortisol level (9) and low ACTH levels (10) are consistent with an increased chance of long-term remission for patients with CD, there are still no accurate models for the preoperative prediction of immediate remission.

ML (machine learning) is a discipline based on computer science and statistics, has the advantage of recognizing relationships between data by learning from datasets iteratively. ML algorithms can be used to predict the outcomes of treatment procedures based on clinical data (11).

In recent years, there have been several studies using ML methods to predict the outcomes of specific diseases. Studies using ML algorithms to predict the recurrence of CD, remission after TSS in patients with acromegaly, response after radiotherapy in patients with acromegaly, and acromegaly patients’ response to somatostatin analogues indicate that the advanced processing power of ML is fit for clinical application (12–14).

Immediate remission is a potential predictive factor for long-term remission and it is important for doctor-patient communication. It is also of great significance to the choice of treatment pathways. The objective of this study was to devise a reliable ML-based model to preoperatively predict immediate remission after TSS in patients with CD.

## Materials and Methods

### Study Population

The current retrospective study was authorized by the ethical review committee of Peking Union Medical College Hospital (PUMCH). All patients hospitalized in PUMCH with a diagnosis of CD between February 2000 and September 2019 were included in the study.

The inclusion criteria were as follows: 1) clinical manifestations of hypercortisolaemia (1); 2) pituitary tumour on magnetic resonance imaging (MRI); 3) exclusion of the possibility of ectopic ACTH syndrome; 4) meeting the criteria for endocrine diagnosis; and 5) surgical and pathological confirmation of tumour. The exclusion criteria were as follows: 1) abortion of surgery; 2) >1 missing value; 3) lack of pathological proof of tumour; 4) lack of record of postoperative prognosis within 7 days (morning (8 am) serum cortisol concentration lower than 5 μg/dl or 24h-UFC lower than 20 μg/d); and 5) incomplete medical records.

In total, 1,045 participants were enrolled in the study and 341 participants were excluded with the exclusion criteria (including 150 pathology-negative patients). All participants were randomly divided into two datasets, a training dataset and a test dataset in a ratio of 8:2.

### Diagnosis of CD

The participants regularly underwent T1-weighted, T2-weighted, T1-weighted gadolinium-enhanced, or dynamic gadolinium-enhanced T1-weighted MRI. In most cases, T1-weighted gadolinium-enhanced MRI was sufficient to demonstrate a suspicion of CD. A pituitary adenoma was suspected if there was a relatively isolated hypointense region within the pituitary gland. In some cases where the profile of the suspected existing tumour was inconspicuous, a dynamic gadolinium-enhanced T1-weighted MRI was needed to outline the tumour and the existence of a tumour would be suspected if there was a relatively hypointense region trailing off over time.

All participants underwent combined low-dose and high-dose dexamethasone suppression tests (LDDST and HDDST) to detect hypercortisolism and identify the location of the tumour, respectively. In the LDDST, a dose of 0.5 mg dexamethasone was given to participants every 6 h for 2 consecutive days, and 24-h urinary free cortisol (24h-UFC) was considered suppressed if it fell below 12.3 μg/24 h on the second day or the plasma cortisol level was lower than 1.8 μg/dl in the morning of the third day. In the HDDST, 2 mg dexamethasone was given to participants every 6 h for 2 consecutive days; 24h-UFC was measured on the second day of drug administration, and plasma cortisol was measured on the morning of the third day. Cortisol was considered to be suppressed if it was reduced by more than 50% compared to the original level. The combination of failure of suppression on the LDDST and successful suppression on HDDST is an indicator of Cushing disease.

The position of the ACTH-secreting adenoma was also confirmed by bilateral petrosal sinus sampling (BIPSS) using desmopressin (instead of CRH) stimulation test in cases where there was uncertainty about the tumour location on preoperative MRI. A 10-mg dose of desmopressin was administered to participants to stimulate ACTH. If the ratio of ACTH concentration in the inferior petrosal sinus to that in peripheral vein (the elbow vein) was greater than 2 in the basal state or greater than 3 after desmopressin stimulation, a diagnosis of CD could be made. The reason that we did not use CRH test in our practice is that CRH is not accessible in China.

The diagnosis of CD was made on the basis of synthesized evidence, including but not limited to MRI scans, combined LDDST and HDDST, BIPSS using desmopressin stimulation test, as well as clinical manifestations, signs, and chief complaints.

All participants underwent TSS by the same two experienced pituitary neurosurgeons (RW and MF). The details of TSS are discussed in our previous study (5). No glucocorticoids or adjuvant medical therapy were given to participants before or during the operation.

### Postoperative Management and Immediate Remission

Plasma cortisol was routinely monitored in the first 3 days after TSS and if the concentration was lower than 5 μg/dl, we initiated glucocorticoid replacement therapy consisting of intravenous hydrocortisone 100 mg two times per day for 3 consecutive days, followed by 30 mg once per day. After being discharged from the hospital, participants decreased their hydrocortisone dosage by 2.5 mg per week, till a dosage of 2.5–5 mg per day. The cessation of glucocorticoid intake was suggested by an endocrinologist or a neurosurgeon after the evaluation of pituitary function.

Immediate remission was defined as morning (8 a.m.) serum cortisol concentration lower than 5 μg/dl or 24-h UFC lower than 20 μg/d (15). Serum cortisol, ACTH and 24-h UFC were all measured with immunoassay method.

### Study Design and ML Algorithms

In ML, data determine the final result and algorithms can only approximate it. Therefore, data preprocessing before model training is particularly important. In the experiment, we performed feature selection and standardized preprocessing on the data.

The selection of features is a process of selecting relevant characteristics and ranking their importance. In our study, we used a univariate feature selection method based on F-test. The F-test, also known as ANOVA, assumes homogeneity of variance and serves as a filtering method to capture the linear relationship between each feature and the label. The highest 1-11 ranked characteristics were sequentially introduced into each ML algorithm. The AUCs of models with different numbers of variables were calculated.

Data standardization can eliminate errors caused by different dimensions or large differences in values. To maintain high prediction performance, we applied min–max normalization to all the data (16). Z-score and non-normalization were also applied, but it turns out that min-max is suitable for normalization to get the model with the highest AUC.

### Algorithms

Five ensemble learning algorithms were applied, namely, the gradient boosting decision tree (GBDT) (17), random forest (RF) (18), adaptive boosting (AdaBoost) (19), extreme gradient boost (XGBoost), and stacking algorithms (20). In addition, 4 non-ensemble learning algorithms, i.e., the logistic regression (LR), naïve bayes (NB) (21), decision tree (DT) (22), and multi-layer perceptron (MLP) algorithms (23) were also used in our experiment. Among these algorithms, LR, NB, and DT are interpretable, but GBDT, RF, AdaBoost, XGBoost, MLP, and stacking are not interpretable, meaning that the functions connecting characteristics and labels are invisible to users. Stacking, which showed the best performance, usually incorporates multiple heterogeneous weak learners, in parallel, combining them by training a “meta-model”, whose inputs are the prediction results of these different weak models. On that basis, the model then outputs a final result.

We applied a total of nine algorithms to predict immediate remission using all 11 variables. First, we selected features according to their rankings from 1 to 11 based on the F-test in the training dataset. Then, we chose the same features in the test dataset. For each iteration, 10-fold cross-validation was applied for training and validation in the training dataset. Later, the grid-search approach (24) was used to select the optimal hyperparameters in each model. Finally, the model that performed best on the validation set was evaluated on the test dataset with the values of area under the receiver operating characteristic curve (AUC), sensitivity (true positive rate), specificity (true negative rate) and Youden’s index. All models were applied and evaluated with the programming language Python version 3.6 (Python Software Foundation of Delaware, USA) and scikit-learn (an ML tool based on the Python language).

### Statistical Analysis

We performed all statistical analyses in Python, version 3.6 (Python Software Foundation, Delaware, United States), RStudio software (1.2.5042) and IBM SPSS Statistics 23 (IBM Corporation). Matplotlib 3.1.3 (comprehensive library for creating static, animated, and interactive visualizations in Python) was used to draw statistical figures. The proportions of missing values for disease duration, body mass index (BMI), tumour size, tumour invasion of the cavernous sinus and combined LDDST and HDDST were 3.9%, 2.8%, 1.7%, 2.6%, and 2.9%, respectively. There were no missing values for any other characteristics. The missing values were imputed with the K-nearest neighbour (KNN) algorithm. The normality of continuous variables was evaluated with the Shapiro-Wilk test. Continuous variables that were normally distributed are displayed as mean ± standard deviation. Continuous variables that were non-normally distributed are displayed as the interquartile range. The Wilcoxon test was used to compare non-normal continuous variables. Categorical variables were displayed as frequencies or percentages and were analysed by the chi-squared test or Fisher’s exact test.

## Results

### Participant Characteristics

In total 1,045 participants treated for CD from February 2000 to September 2019 were enrolled in this study.

The values of the 11 predictors in all participants (836 from the training dataset and 209 from the test dataset) are shown in **Table 1**. A total of 766 participants (73.3%) exhibited immediate remission postoperatively. No obvious interclass differences were found in gender, age, first operation, disease duration, BMI, 24-h preoperative UFC (P24h-UFC),combined LDDST and HDDST (combined LH), preoperative morning serum cortisol (PMS-C), preoperative ACTH, tumour size (microadenoma or macroadenoma) or cavernous sinus invasion evaluated on MRI (IOMRI). Microadenoma was defined as the largest diameter of the tumour <10 mm, and macroadenoma was defined as the largest diameter ≥10 mm. IOMRI was defined as Knosp 3 or Knosp 4 adenoma on MRI. The characteristics of patients in remission and non-remission groups are shown in **Table 2**.

**Table 1:** Participants’ characteristics in training and test datasets.

Characteristic	Total	Training dataset	Test dataset	P-value
**N**	1045	836	209	
**Gender**				
Male	201(19.23%)	168(20.10%)	33(15.79%)	0.158
Female	844(80.77%)	668(79.90)	176(84.21%)	
**Age(year)**	35(26–45)	35(26–45)	34(26–44)	0.858
**First operation**				
Yes	925(88.52%)	740(88.52%)	185(88.52%)	1.000
No	120(11.48%)	96(11.48)	24(11.48%)	
**Disease duration(month)**	46(24–72)	46(21–73)	46(24–72)	0.883
**BMI(kg/m²)**	25.96(23.76–28.60)	25.96(23.75–28.65)	26.10(23.82–28.39)	0.886
**P24h-UFC(μg/d)**	467.52(279–791.7)	467.61(278.95–790.85)	466.9(283.6–812.8)	0.845
**Combined LDDST and HDDST**				
Positive	856(81.91%)	679(81.22%)	177(84.69%)	0.244
Negative	189(18.09%)	157(18.78%)	32(15.31%)	
**preoperative ACTH(pg/ml)**	71.26(48.2–110)	71.10(47.88–111.00)	72.60(50.90–109.00)	0.748
**PMS-C(μg/dl)**	26.38(21.46–32.12)	26.31(21.34–31.96)	26.61(21.86–33.40)	0.589
**Tumour size**				
microadenoma	845(80.86%)	679(81.22%)	166(79.43%)	0.555
macroadenoma	200(19.14%)	157(18.78%)	43(20.57%)	
**IOMRI**				
Positive	66(6.32%)	50(5.98%)	16(7.66%)	0.373
Negative	979(93.68%)	786(94.02%)	193(92.34%)	
**Prognosis**				0.828
remission	766(73.30%)	613(73.33%)	153(73.21%)	
non-remission	279(26.70%)	223(26.67%)	56(26.79%)	

BMI, body mass index; P24h-UFC, preoperative 24-h urinary free cortisol; PMS-C, preoperative morning (8 a.m.) serum cortisol; IOMRI, invasion of cavernous sinus on preoperative MRI.

**Table 2:** Patients’ characteristics in remission and non-remission groups.

Characteristic	remission	nonremission	p-value
**Gender**	766	279	
Male	143(18.67%)	58(20.79%)	0.442
Female	623(81.33%)	221(79.21%)	
**Age(year)**	35(27–45)	33(25–43)	0.170
**First operation**			
Yes	700(91.38%)	225(80.65%)	<0.001
No	66(8.62%)	54(19.35%)	
**Disease duration(month)**	39(22–72)	48(24–84)	0.010
**BMI(kg/m²)**	25.90(23.65–28.45)	26.43(24.02–29.09)	0.146
**P24h-UFC(μg/d)**	459.1(287.6–793.3)	482.4(258.0–796.5)	0.830
**Combined LDDST and HDDST**			
Positive	634(82.77%)	222(79.57%)	0.235
Negative	132(17.23%)	57(20.43%)	
**PMS-Cortisol(μg/dl)**	26.44(21.34–32.19)	26.07(21.86–31.97)	0.840
**preoperative ACTH(pg/ml)**	68.3(47.1–108.0)	80.0(51.0–119.0)	0.008
**Tumour size**			
Micro	646(84.33%)	199(71.33%)	<0.001
Macro	120(15.67%)	80(28.67%)	
**IOMRI**			
Yes	30(3.92%)	36(12.90%)	<0.001
No	736(96.08%)	243(87.10%)	

BMI, body mass index; P24h-UFC, preoperative 24-h urinary free cortisol; combined low-dose and high-dose dexamethasone suppression test; PMS-C, preoperative morning (8 a.m.) serum cortisol; IOMRI, invasion of cavernous sinus on preoperative MRI.

### Predictive Performance of the Nine Models

**Figure 1:**
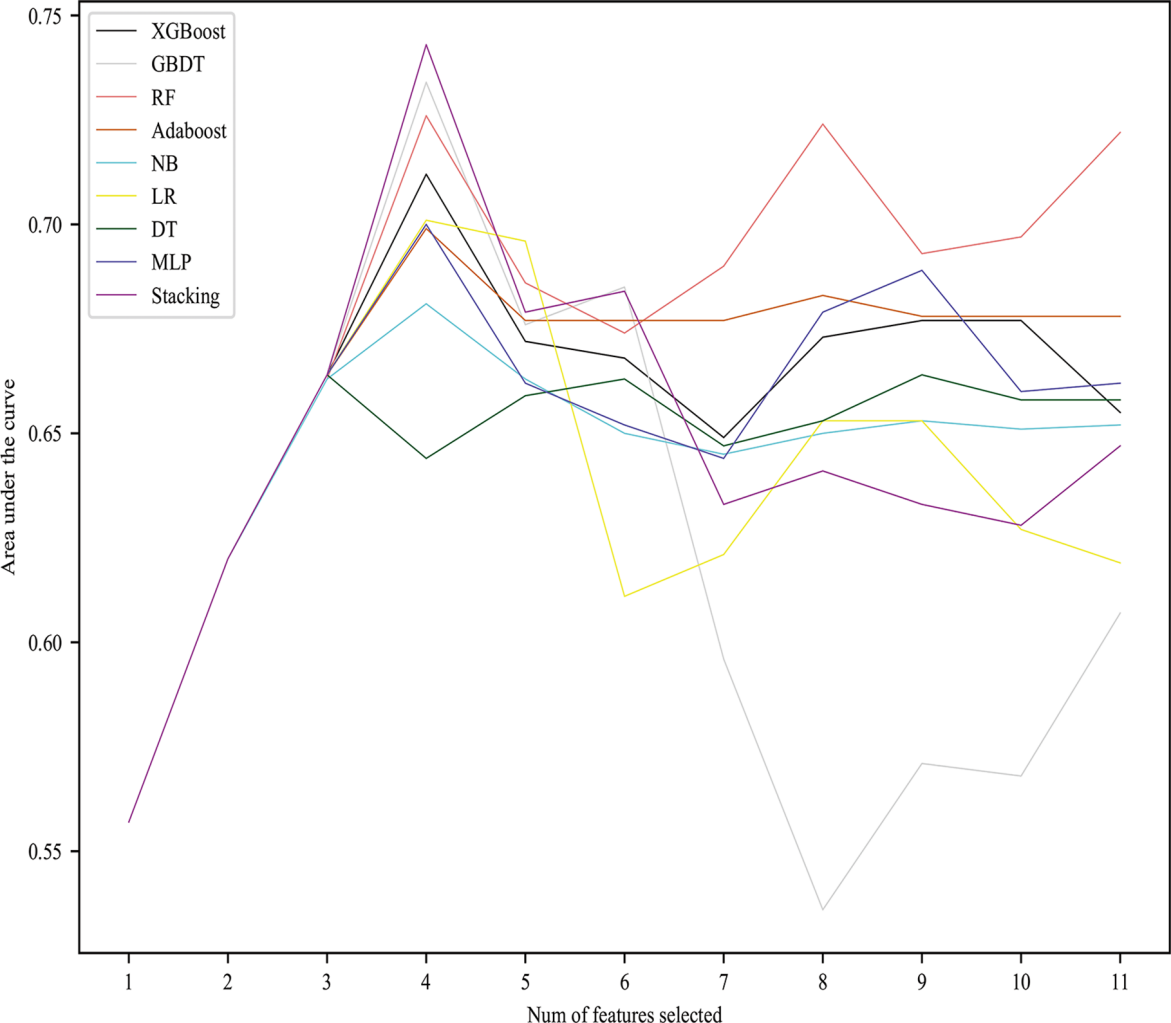
AUC values of nine models with different numbers of features selected.

**Figure 2:**
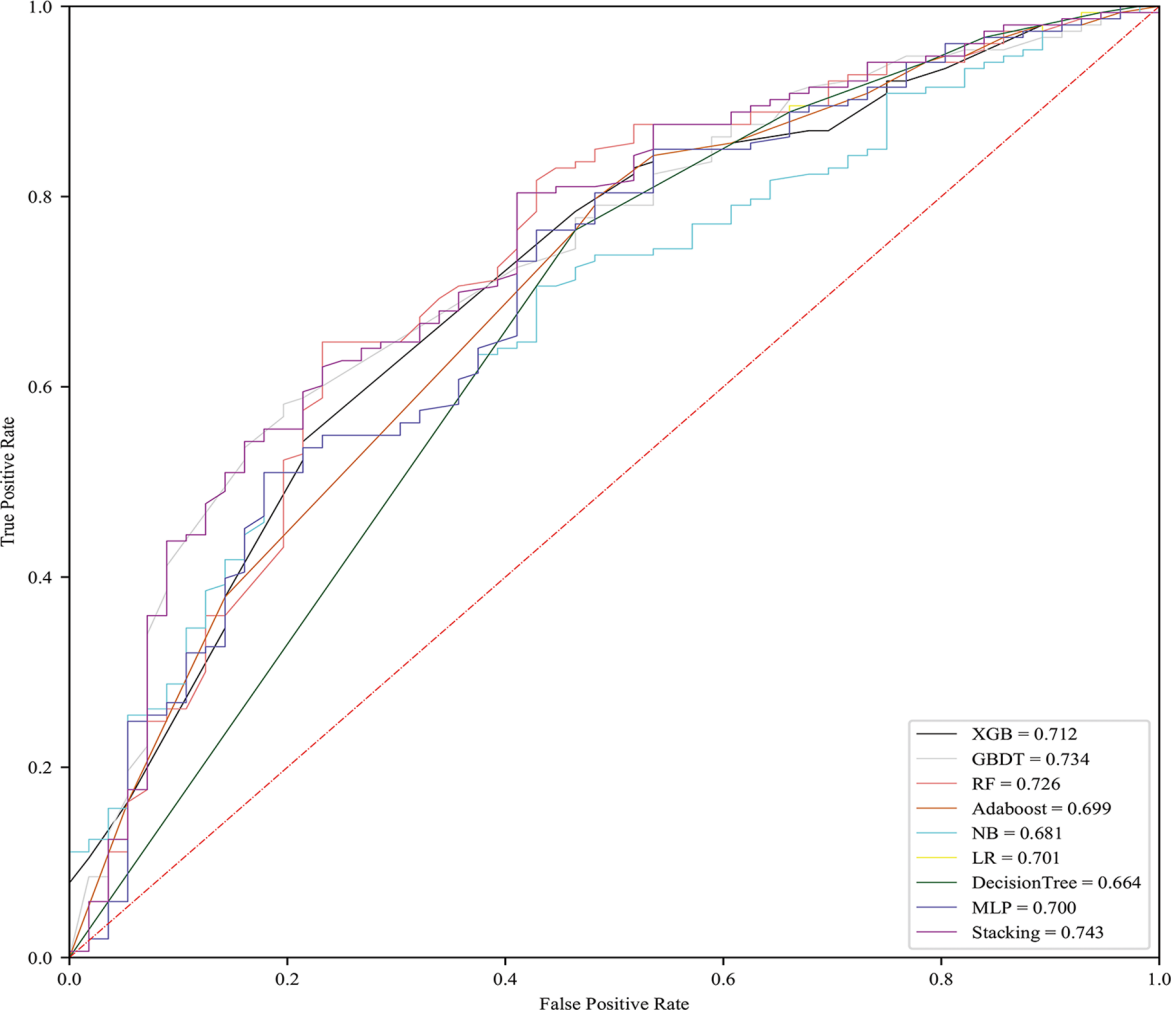
Performances of models with optimal number of features.

To clarify the relationship between predictive performance and the number of characteristics selected in the model, we introduced all features in order of importance to 9 algorithms. We found that, as the number of selected characteristics increased, the prediction performance of the model initially improved, but once the optimal number of features was reached, the performance declined if the number of selected characteristics was further increased. All models except DT performed best when the number of features is 4, achieving a much greater AUC than it when all features were used (**Figure 1**). **Figure 2** shows the performance of each model at the optimal number of features. The best predictive performance was achieved by the stacking algorithm (AUC = 0.743, 95% confidence interval (0.677,0.806), ACC=0.746) which outperformed GBDT (AUC = 0.734), RF (AUC = 0.726), LR (AUC = 0.701), XGB (AUC = 0.712), MLP (AUC = 0.700), and AdaBoost (AUC = 0.699) and significantly outperformed NB (AUC = 0.681) and DT (AUC = 0.664). The outcomes of different models are shown in **Table 3**.

**Table 3:** Highest AUCs of different models.

Algorithm	AUC	95% confidence interval	Accuracy
Extreme Gradient Boost	0.712	0.645–0.776	0.732
Gradient Boosting Decision Tree	0.734	0.669–0.796	0.732
Random Forest	0.726	0.656–0.792	0.746
Adaptive Boost	0.699	0.632–0.764	0.737
Naïve Bayes	0.681	0.614–0.744	0.684
Logistic regression	0.701	0.630–0.767	0.732
Decision Tree	0.664	0.598–0.726	0.742
Multi-layer Perceptron	0.700	0.629–0.766	0.732
Stacking	0.743	0.677–0.806	0.746

AUC, area under the curve; Accuracy, the percentage of participants whose postoperative immediate prognoses were correctly predicted with the ML-based model among the cohort.

The highest Youden’s index for stacking was 0.393, and the sensitivity and specificity were 80.4% and 58.9%, respectively.

### Variable Importance

**Figure 3:**
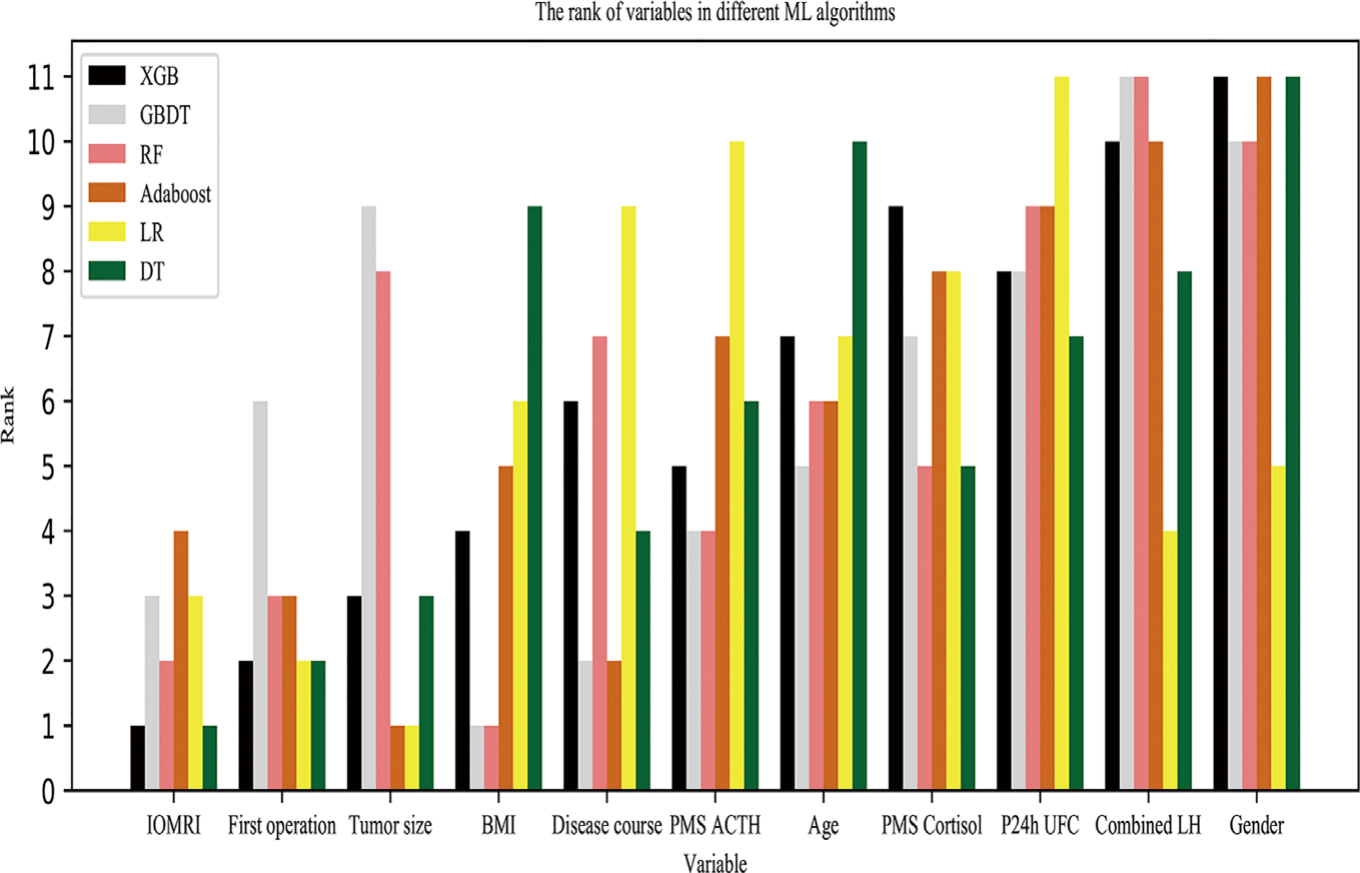
Ranking of variables by six models. The six models output their own ranking of variable importance.

**Figure 4:**
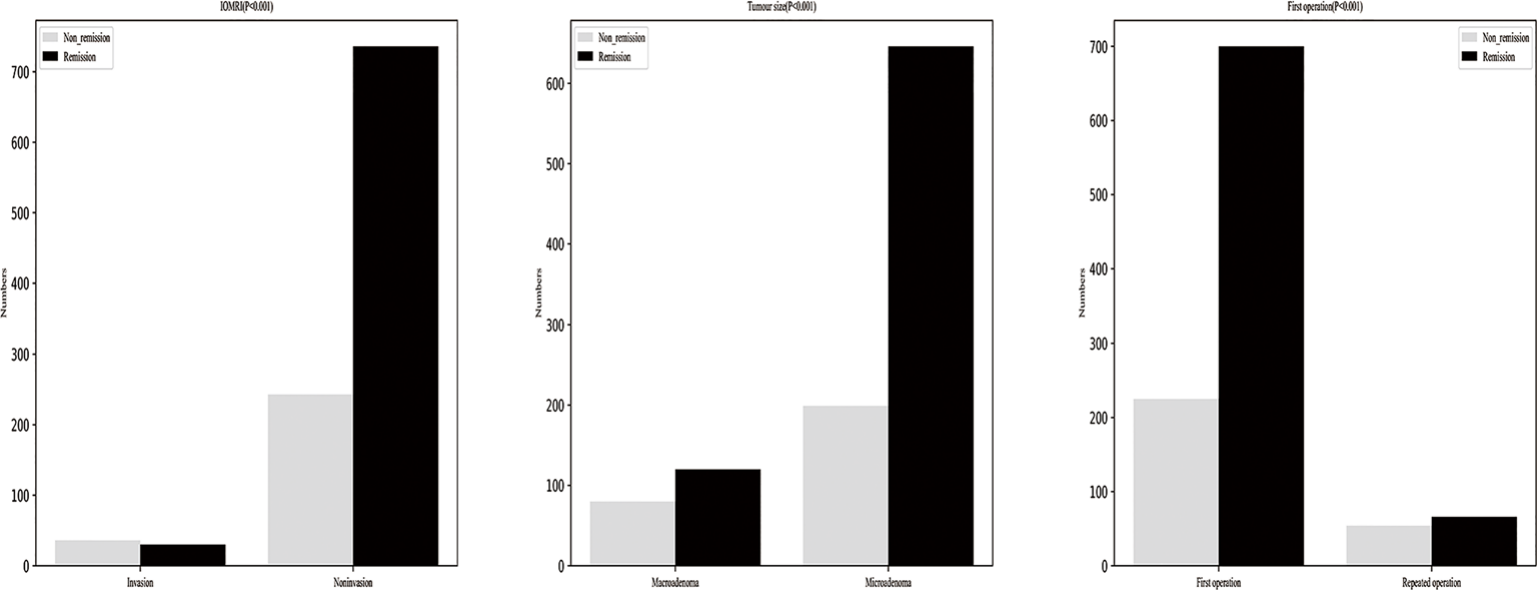
Remission rates’ difference in different groups divided by IOMRI, tumour size and operation times.

We selected the best features through a statistical test based on F-test univariate analysis, and the features were ranked for importance as follows, IOMRI, tumour size, first operation, preoperative ACTH, disease duration, combined LH, BMI, age, P24hUFC, gender, PMS-C. Six models also output their own variable importance rankings, and the rankings are shown in **Figure 3**. The top 3 most important predictors were IOMRI, first operation and tumour size. For the top 3 most important predictors, we also performed chi-squared analysis to evaluate the remission rates in different groups (**Figure 4**).

## Discussion

TSS is the first-line treatment for CD by neurosurgeons. According to a previous study by Ioachimescu AG et al., the immediate remission rate ranges between 59% and 96.6% (25). In the present cohort, the overall immediate remission rate was 73.3%, which approximates the 76% remission rate reported by a meta-analysis (26). According to a recent study, immediate remission may be a compelling predictor of long-term remission (6). Therefore, it is important to develop a preoperative prediction model for immediate remission.

To our knowledge, this is the largest cohort and first ML-based model for the preoperative prediction of immediate remission after TSS. Conventional statistical approaches have usually been the first choice in previous studies. In a previous study by our team (27), conventional biostatistical methods were used to detect the preoperative predictors for immediate remission in patients with Cushing’s disease after TSS. The AUC of receiver operating characteristic curve for single preoperative ACTH predicting immediate remission is 0.553 (cutoff value=67.35 ng/L, sensitivity 60.9%, specificity 49.5%). Disease duration ≤64.5 months predicted immediate remission with an AUC of 0.552 (40.5% sensitivity and 71.0% specificity). The AUC value(0.743) of the present ML-based model is conspicuously higher than the ones(0.553 and 0.552) in our previous study which indicates that the ML-based model can more accurately predict immediate remission.

The ML-based model is completely different from conventional methods in that it can learn the patterns of health trajectories from electronic health datasets themselves. ML models may help clinicians to gain knowledge from a vast quantity of data far beyond any individual clinician’s experience (28). The result from ML methods has its own scalability and flexibility advantages over traditional statistics. In our analysis, therefore, an ML-based method including nine algorithms was applied, leading us to the conclusion that a model constructed with IOMRI, tumour size, first operation and preoperative ACTH had the best performance (AUC=0.743).

We developed an online calculator to facilitate the prediction which can output the probability of immediate remission with input of preoperative factors (http://smk921101.pythonanywhere.com/index). With the output from the calculator, surgeons can talk to patients about the possible prognosis. Thus, the ML-based model is important in providing information about prognosis and doctor–patient communication.

There are advantages as well as disadvantages of ML algorithms. Simple statistical method such as single-factor logistic regression is a kind of simple algorithm of ML as well as a traditional statistical method. It is a linear classifier, so it cannot handle the correlation between features. At the same time, the performance may be poor (AUC) and the accuracy may be low. In contrast, we used a variety of ensemble learning methods in our experiment. The idea is to combine multiple weak learners into one strong learner. The advantage of ensemble learning is to ensure the diversity of weak classifiers, so that the results obtained are often better than those of a single learner. There are also some disadvantages for ML-based models. On the one hand, ML requires enough data to train the model, otherwise it will easily lead to overfitting. Our study presented the largest cohort which is beneficial for the analysis of ML method. On the other hand, some ML methods have poor interpretability, thus it needs an independent test dataset. We divided the cohort into a training dataset and an independent test dataset, but it would be better to include data from another centre as a test dataset in the future.

In the present study, the model with the best performance showed that IOMRI was ranked as the top characteristic related to immediate remission which is consistent with previous studies and our experience (29). Tumour size is ranked as the second most important characteristic. Patients with microadenomas tended to have a higher immediate remission rate than patients with macroadenomas, which is consistent with a previous study (26). Similarly, other studies also found that patients with macroadenomas tended to have lower immediate remission rates than patients with microadenomas after TSS (30, 31). Patients who have a history of TSS is ranked as the third most important characteristic. Patients with a history of TSS tended to have an increased probability of non-remission in our study, which is in accordance with previous studies (32, 33). This may be because a second TSS often indicates a more invasive and aggressive behaviour of the tumour in which there may be a residual tumour after TSS. Preoperative ACTH is ranked as the fourth most important predictor. The four predictors mentioned above were included in the stacking algorithm with highest AUC.

Disease duration was not included in the final ML-based model, but it was ranked as the fifth most important predictor and had obvious significance in univariate analysis. The results of a study by Elena Y et al. showed that the disease duration is not correlated with the recurrence of CD (34). Thus, the actual relationship between disease duration and immediate remission remains to be investigated in a larger or multicentre cohort. In a previous study, Jessica K et al. reported that older age was a strong predictor of mortality following TSS (35), but in other studies, age was not correlated with recurrence (6, 36). In our study, age was not included in the final model. And the model also excluded the combined LDDST and HDDST results and BMI which were not significantly correlated with immediate remission according to univariate analysis. There have been several noteworthy contradictions among different studies investigating predictors of remission and these differences may be attributable to the small cohorts and difficulty in unveiling the complex relationships of the vast number of variables with conventional biostatistical methods. This is exactly where the advantages of ML lie. We believe that an ML-based model may be more effective than previous methods as a way to understand the disease in the future.

## Limitations and Strengths

The present study has some limitations. First, the clinical data still had some missing values. Second, a larger cohort would be more suitable for ML analysis, although, to our knowledge this study already has the largest CD cohort in the literature. Third, this is a single centre study. However, our cohort is larger than most cohorts in previous studies and immediate remission was evaluated accurately. One strength of this study is that it is the first to carry out preoperative prediction of immediate remission using ML methods, whose performance is better than that of traditional methods, such as LR and NB.

## Conclusion

TSS is the preferred choice of neurosurgeons to treat CD, and ML-based models can be used to preoperatively predict immediate remission. In this study, stacking algorithm outperformed the traditional ones, such as LR and NB. Invasion of cavernous sinus on MRI, the largest diameter of tumour, first operation, and preoperative ACTH were included in the final model.

## Data Availability Statement

Some or all datasets generated and/or analysed during the current study are not publicly available but are available from the corresponding author on reasonable request. If the datasets are needed, the corresponding author RW would be in contact.

## Ethics Statement

The studies involving human participants were reviewed and approved by ethical review committee of Peking Union Medical College Hospital (PUMCH). Written informed consent to participate in this study was provided by the participants’ legal guardian/next of kin.

## Author Contributions

WZ, MS, and YF contributed equally to the present study. Each author contributes to the article in data collecting and analysis. RW, SZ, and MF take final responsibility for this article. All authors contributed to the article and approved the submitted version.

## Funding

This work was supported by the Graduate Innovation Fund of Peking Union Medical College (2018-1002-01-10) and by the, Natural Science Foundation of Beijing Municipality (grant number 7182137).

## Conflict of Interest

The authors declare that the research was conducted in the absence of any commercial or financial relationships that could be construed as a potential conflict of interest.

## Abbreviations

ML, machine learning; TSS, transsphenoidal surgery; CD, Cushing’s disease; ROC, receiver operating characteristic curve; AUC, area under the curve; UFC, urine free cortisol; LDDST, low-dose dexamethasone suppression test; HDDST, high-dose dexamethasone suppression test; MRI, magnetic resonance imaging; BIPSS, bilateral inferior petrosal sinus sampling; PMS-C, preoperative morning (8 a.m.) serum cortisol; P24h-UFC, preoperative 24-h urinary free cortisol; LR, logistic regression; GBDT, Gradient Boosting Decision Tree; XGBoost, Extreme Gradient Boost; AdaBoost, Adaptive Boosting; RF, random forest; NB, naïve bayes; DT, decision tree; MLP, multi-layer perception; BMI, body mass index; IOMRI, invasion of cavernous sinus on preoperative MRI.
