# Supplementary material for: Machine Learning in Preoperative Prediction of Postoperative Immediate Remission of Histology-Positive Cushing’s Disease
Source: Front Endocrinol (Lausanne). 2021 Mar 2;12:635795. doi: 10.3389/fendo.2021.635795 (PMC7961560; doi:10.3389/fendo.2021.635795)
Supplement: Supplementary file 1 [file Table_1.docx]

**Supplemental Table. Algorithms’ hyperparameters**

| Algorithm | Parameters |
| --- | --- |
| Extreme Gradient Boost | XGBClassifier(base_score=0.5, booster=None, colsample_bylevel=1,colsample_bynode=1, colsample_bytree=1, gamma=0, gpu_id=-1,  importance_type='gain', interaction_constraints=None,learning_rate=0.3, max_delta_step=0, max_depth=2,min_child_weight=10, missing=nan, monotone_constraints=None,n_estimators=16, n_jobs=-1, num_parallel_tree=1,objective='binary:logistic', random_state=0, reg_alpha=6,reg_lambda=1, scale_pos_weight=1, subsample=1, tree_method=None,validate_parameters=False, verbosity=None) |
| Gradient Boosting Decision Tree | GradientBoostingClassifier(ccp_alpha=0.0,criterion='friedman_mse', init=None,learning_rate=0.1, loss='deviance', max_depth=3,max_features=None, max_leaf_nodes=None,  min_impurity_decrease=0.0, min_impurity_split=None, min_samples_leaf=4, min_samples_split=12, min_weight_fraction_leaf=0.0,n_estimators=17,n_iter_no_change=None, presort='deprecated',random_state=0, subsample=0.9, tol=0.0001, validation_fraction=0.1, verbose=0,warm_start=False) |
| Random Forest | RandomForestClassifier(bootstrap=True, ccp_alpha=0.0, class_weight=None,criterion='gini', max_depth=4, max_features='auto',  max_leaf_nodes=15, max_samples=None,min_impurity_decrease=0.0, min_impurity_split=None, min_samples_leaf=2, min_samples_split=49, min_weight_fraction_leaf=0.0, n_estimators=114,n_jobs=-1, oob_score=False, random_state=0, verbose=0, warm_start=False) |
| Adaptive Boost | AdaBoostClassifier(algorithm='SAMME.R',base_estimator=DecisionTreeClassifier(ccp_alpha=0.0,class_weight=None,criterion='gini',max_depth=1,max_features=None,max_leaf_nodes=None,min_impurity_decrease=0.0,min_impurity_split=None,min_samples_leaf=20,min_samples_split=2,min_weight_fraction_leaf=0.0,presort='deprecated',random_state=0,splitter='best'),learning_rate=0.1,n_estimators=34, random_state=0) |
| Naïve Bayes | default |
| Logistic regression | LogisticRegression(C=0.030688378919176425,class_weight=None,dual=False,fit_intercept=True,intercept_scaling=1,l1_ratio=None,max_iter=100,multi_class='auto',n_jobs=None,penalty='l2',random_state=0,solver='liblinear',tol=0.0001,verbose=0,warm_start=False) |
| Decision Tree | DecisionTreeClassifier(ccp_alpha=0.0, class_weight=None, criterion='gini', max_depth=3, max_features=None, max_leaf_nodes=None, min_impurity_decrease=0.0, min_impurity_split=None, min_samples_leaf=2, min_samples_split=5, min_weight_fraction_leaf=0.0, presort='deprecated', random_state=0, splitter='random') |
| Multi-layer Perceptron | MLPClassifier(activation='tanh', alpha=1, batch_size='auto', beta_1=0.9, beta_2=0.999, early_stopping=False, epsilon=1e-08, hidden_layer_sizes=(85, 38), learning_rate='constant', learning_rate_init=0.001, max_fun=15000, max_iter=1000, momentum=0.9, n_iter_no_change=10, nesterovs_momentum=True, power_t=0.5, random_state=None, shuffle=True, solver='sgd', tol=0.0001, validation_fraction=0.1, verbose=False, warm_start=False) |
| Stacking | StackingClassifier(classifiers=[XGB,GBDT,RF,Adaboost,NB,LR,DT,MLP],meta_classifier= LogisticRegression(),use_probas=True,drop_last_proba=True, average_probas=False,use_features_in_secondary=False) |
